# MEOX1 Inhibits Growth and Metastasis of Salivary Adenoid Cystic Carcinoma

**DOI:** 10.3390/cimb48050485

**Published:** 2026-05-06

**Authors:** Huaxiu Sun, Yuping Liu, Yajuan Cui, Zheng Zhou, Zhanlan Wu, Chuan-Xiang Zhou

**Affiliations:** Department of Oral Pathology, Peking University School and Hospital of Stomatology, National Center of Stomatology, National Clinical Research Center for Oral Disease, National Engineering Research Center of Oral Biomaterials and Digital Medicine Devices, Beijing 100081, China

**Keywords:** MEOX1, salivary adenoid cystic carcinoma, metastasis, cell cycle arrest

## Abstract

Salivary adenoid cystic carcinoma (SACC) is a malignant salivary gland neoplasm characterized by aggressive local invasion and a marked propensity for metastasis. However, the role of MEOX1 in SACC progression remains poorly defined. In this study, we examined the effects of MEOX1 overexpression on the malignant behavior of SACC cells in vitro and in vivo. Human SACC-83 and SACC-LM cells were transduced with lentiviral vectors encoding MEOX1 or an empty vector control, and cell proliferation, migration, invasion, and cell cycle distribution were assessed using CCK-8, wound healing, Transwell, and flow cytometric assays, respectively. RNA sequencing was performed to characterize transcriptional changes associated with MEOX1 overexpression. In vivo, tumor growth was evaluated in BALB/c nude mice bearing subcutaneous xenografts, and pulmonary metastatic colonization was assessed using a tail vein injection model. MEOX1 overexpression reduced the proliferation, migration, and invasion of SACC cells in vitro and increased the G2/M phase fraction. In xenograft models, MEOX1-overexpressing cells formed smaller tumors and showed lower Ki67 staining than control cells. In the experimental lung metastasis model, mice injected with MEOX1-overexpressing cells developed fewer pulmonary metastatic nodules. RNA-seq identified 588 differentially expressed genes associated with MEOX1 overexpression, with enrichment in pathways including cytokine–cytokine receptor interaction, Toll-like receptor signaling, and G protein-coupled receptor signaling. Together, these findings indicate that enforced MEOX1 expression is associated with reduced malignant phenotypes in SACC models and with transcriptomic alterations in pathways related to immune response, G protein-coupled receptor signaling, and DNA damage response.

## 1. Introduction

Salivary adenoid cystic carcinoma (SACC) accounts for approximately 10% of all salivary gland cancers. It is characterized by a marked propensity for perineural invasion and distant dissemination, most commonly to the lungs, with pulmonary metastases representing the predominant metastatic pattern in affected patients [1]. Lung metastasis has been reported in up to 70% of cases and remains a major determinant of long term outcomes [2]. Although surgery with adjuvant therapy remains the mainstay of treatment, clinical outcomes are still unsatisfactory, largely because of local invasiveness, late recurrence, and distant metastatic spread [3,4]. A more complete understanding of the molecular programs that underlie SACC progression is therefore needed. In particular, the biological basis for the paradoxical coexistence of relatively indolent growth and strong invasive and metastatic potential remains incompletely defined [5]. In addition to oncogenic drivers, emerging evidence suggests that disruption of suppressive regulatory mechanisms may also contribute to SACC progression. Studies of SACC have implicated chromosome level loss, tumor-suppressive microRNAs, and phosphatase signaling abnormalities, indicating that multiple layers of growth restraint may be compromised in this disease. However, whether transcription-associated regulators participate in this broader suppressive landscape remains largely unclear.

Mesenchyme homeobox 1 (MEOX1), located on human chromosome 17q21, was initially identified in 1994 as a gene associated with the BRCA1 locus [6]. MEOX1 plays essential roles in embryogenesis, including mesoderm specification, somite development, and cardiovascular morphogenesis [7]. Its expression during development has been documented in craniofacial tissues, the eye, heart, kidney, and pharyngeal arches, consistent with its role in musculoskeletal patterning and organogenesis [8,9]. In pathological contexts, MEOX1 has been implicated in fibrotic remodeling, including cardiac and pulmonary fibrosis, where it contributes to fibroblast activation downstream of TGF-β1 signaling [10,11].

Recent studies further suggest that MEOX1 may influence tumor progression, although its function appears to vary across tumor types. In breast and ovarian cancers, MEOX1 has been linked to tumor growth and invasion, whereas in non-small-cell lung cancer, it has been proposed to suppress malignant behavior through the regulation of cell cycle machinery, including CCNB1 [12,13,14]. These findings point to a context-dependent role for MEOX1 in cancer. However, whether MEOX1 contributes to the biology of salivary gland tumors, particularly SACC, remains unknown. This makes MEOX1 a potentially relevant but underexplored candidate for understanding how transcriptional regulation may contribute to SACC progression and metastatic behavior.

In this study, we aimed to examine the phenotypic and transcriptomic changes associated with MEOX1 overexpression in SACC using both in vitro and in vivo models. Through gain-of-function assays and transcriptomic analyses, we examined the associations of MEOX1 overexpression with tumor cell proliferation, cell cycle distribution, invasion, and metastatic behavior.

## 2. Materials and Methods

### 2.1. Cell Culture

The human SACC cell lines SACC-83 and SACC-LM were obtained from the Peking University School of Stomatology. SACC-83 cells were cultured in RPMI-1640 medium (Gibco, Thermo Fisher Scientific, Grand Island, NY, USA) supplemented with 10% fetal bovine serum (FBS; Gibco, Thermo Fisher Scientific, São Paulo, Brazil), while SACC-LM cells were cultured in DMEM/F12 medium (Gibco, Thermo Fisher Scientific, Grand Island, NY, USA) with 10% FBS. All cells were incubated in a humidified atmosphere at 37 °C with 5% CO2. Cell line identity was authenticated by short tandem repeat (STR) profiling (BioWing, Shanghai, China), and all cell lines were confirmed to be free of mycoplasma contamination using a Mycoplasma Detection Kit (ABclonal, Wuhan, China, Cat. No. RK0043).

### 2.2. Vector Construction and Lentivirus Production

The human MEOX1 coding sequence was inserted into the lentiviral expression vector pGMLV-CMV-H_MEOX1-3×Flag-PGK-Puro to generate the overexpression construct (OE-MEOX1), which encoded MEOX1 with a 3×Flag tag and a puromycin resistance cassette for stable selection. Lentiviral particles were produced in 293FT cells. Briefly, 293FT cells were seeded in 6 cm dishes and cultured to 70–80% confluence. Before transfection, the culture medium was replaced with 3.8 mL Opti-MEM for approximately 30 min. A transfection mixture containing 6 μg expression plasmid, 3 μg pMD2.G, 3 μg psPAX2, and 24 μL Lipofectamine 2000 (Invitrogen, Thermo Fisher Scientific, Carlsbad, CA, USA) was prepared in Opti-MEM, incubated at room temperature for 15–20 min, and then added dropwise to the cells. After 4–6 h, the transfection medium was replaced with 5 mL DMEM supplemented with 30% FBS. Viral supernatants were collected at 48 h, filtered through a 0.45 μm membrane, and stored at 4 °C for short-term use or at −80 °C for long-term preservation.

### 2.3. Viral Infection and Cell Selection

SACC cells were seeded in 6-well plates and infected when cell confluence reached approximately 50%. For each well, the original medium was removed and replaced with a mixture of 1.5 mL fresh complete medium and 500 μL lentiviral supernatant, together with 6–8 μg/mL polybrene to enhance transduction efficiency. After 8–12 h, the viral medium was replaced with routine culture medium. A puromycin kill curve was performed before stable selection, and 1.0 μg/mL puromycin was used as the final selection concentration. Stable cell lines were obtained after 3–7 days of selection and were validated by Western blot analysis. After parental cells were eliminated, surviving cells were expanded and cryopreserved.

### 2.4. Western Blot Analysis

Protein samples were mixed with 5× loading buffer at a ratio of 4:1, heated at 99 °C for 5 min, and separated by SDS-PAGE using 30 μg total protein per lane. The separated proteins were then transferred onto nitrocellulose membranes, which were blocked with blocking buffer (NCMbio, Suzhou, China) and incubated overnight at 4 °C with the following primary antibodies: anti-FLAG (Proteintech, Wuhan, China, Cat. No. 20543-1-AP, 1:2000) and anti-Vinculin (Proteintech, Cat. No. 66305-1-Ig, 1:2000). After washing with TBST, membranes were incubated for 1 h at room temperature with the appropriate HRP-conjugated secondary antibodies, including goat anti-mouse IgG (Proteintech Cat. No. SA00001-1, 1:5000, China) and goat anti-rabbit IgG (Proteintech Cat. No. SA00001-2, 1:5000, China). Protein bands were visualized using an ultrasensitive ECL chemiluminescence kit (NCMbio, Suzhou, China), and the signals were captured using an e-BLOT imaging system Thermo Fisher Scientific, Waltham, MA, USA).

### 2.5. Cell Counting Kit-8 (CCK-8)

Cell proliferation was quantified using a CCK-8 assay kit (ApexBio Technology, Houston, TX, USA). Cells were seeded at a density of 2 × 10^3^ cells/well in 96-well plates containing 100 μL of complete medium. At designated time points, 10 μL of CCK-8 reagent was added, and the absorbance at 450 nm was measured after 2 h using a microplate reader (BioTek, Winooski, VT, USA). The assay was performed using three independent biological replicates, with five technical replicates for each condition in each experiment. Data were analyzed using GraphPad Prism(10.3.0).

### 2.6. Migration and Invasion Assay

For migration assays, 7 × 10^4^ cells were resuspended in serum-free medium and seeded in the upper chamber of 24-well Transwell inserts with an 8 μm pore size (Corning, Kennebunk, ME, USA). For invasion assays, inserts were precoated with Matrigel (6 μL Matrigel in 54 μL medium; BD Biosciences, San Jose, CA, USA) and incubated for 6 h, and cells were resuspended in serum-free medium before seeding into the upper chamber. Medium containing 10% FBS was added to the lower chamber as a chemoattractant. After 24 h, cells that had migrated or invaded were fixed, stained, and counted in three random microscopic fields under a microscope (Olympus, Tokyo, Japan). Migration and invasion assays were performed using three independent biological replicates. Data were analyzed using GraphPad Prism.

### 2.7. Wound Healing Assay

Cells were cultured to confluence in 6-well plates and serum-starved for 24 h before scratching. A linear scratch was introduced using a sterile pipette tip, followed by washing with PBS to remove debris. Cells were cultured in serum-free medium and imaged at 0 and 24 h using an inverted microscope. Migration rates were calculated as (Wound area at 0 h − wound area at 24 h)/wound area at 0 h × 100%. Wound healing assays were performed using three independent biological replicates.

### 2.8. Cell Cycle Detection

Cells were collected by trypsinization, washed with PBS, and centrifuged at 1200 rpm for 10 min. Cell pellets were fixed with pre-chilled 70% ethanol at 4 °C overnight. After washing with PBS, cells were stained with PI/RNase A solution at a 9:1 ratio for 30–60 min in the dark at room temperature. Red fluorescence was detected at an excitation wavelength of 488 nm by flow cytometry. For analysis, the main cell population was first selected based on forward- and side-scatter properties to exclude debris and non-cellular events, followed by further gating to exclude cell aggregates before cell cycle modeling. The final gated population was analyzed using ModFit LT (Version 5.0) to determine cell cycle distribution, and statistical analysis was conducted in SPSS (Version 27.0).

### 2.9. In Vivo Experiments

Six-week-old female BALB/c nude and NOD/SCID mice (SPF, GemPharmatech, Beijing, China) were maintained under specific pathogen-free conditions with free access to food and water. All procedures were approved by the Institutional Animal Care and Use Committee (IACUC) of Peking University Health Science Center (Approval Number: DLAS-BE0385; Approval Date: 20 March 2025) and conducted in accordance with the ARRIVE 2.0 guidelines and the 3Rs principle.

For subcutaneous tumor growth assays, BALB/c nude mice (*n =* 6 per group) were randomly assigned to control or MEOX1-overexpressing groups using a computer-generated random number sequence. Mice were injected subcutaneously with 5 × 10^6^ SACC-LM cells (OE-MEOX1 or vector) in a 1:3 PBS/Matrigel mixture. No anesthesia was required for this procedure because the injection was minimally invasive and caused no observable distress. Tumor size and body weight were measured on days 3, 7, 11 and 15 by investigators blinded to group assignment. Tumor volume was calculated using the formula (Length × Width^2^)/2.

For the experimental metastasis model, NOD/SCID mice were injected via their tail vein with 5 × 10^6^ SACC-LM cells (OE-MEOX1 or vector) without anesthesia, as the procedure was brief and non-invasive. During the 8-week observation, one mouse per group died of non-tumor causes and was excluded from analysis. Five mice per group were included in the final analysis. Each mouse was considered an independent biological replicate.

Mice were monitored daily for general health, and humane endpoints were predefined according to IACUC guidelines. At the experimental endpoint, all surviving mice were euthanized via cervical dislocation under IACUC approved protocols, and lung tissues were harvested, fixed in formalin, and subjected to histological analysis.

### 2.10. IHC Staining

Formalin-fixed, paraffin-embedded tumor tissues were sectioned at a 4 μm thickness and subjected to standard IHC protocols. Primary antibodies included MEOX1 (Abcam, Cambridge, UK, ab105349, 1:200), Ki67 (Cell Signaling Technology, Beverly, MA, USA, 9449, 1:200), and pan-CK (ZSGB-Bio, Beijing, China, AM-0069, 1:200). IHC staining was independently evaluated by two blinded pathologists. Only tumor cells were scored. Immunostaining was semi-quantitatively assessed according to the percentage of positively stained tumor cells as follows: 0 (0–1%), 1 (2–25%), 2 (26–50%), 3 (51–75%), and 4 (>75%). Group comparisons of IHC scores were performed using the Mann–Whitney U test.

### 2.11. RNA-Seq

Total RNA was isolated using TRIzol reagent (Invitrogen, USA). RNA quantity and purity were assessed using a NanoDrop ND-1000 spectrophotometer, and RNA integrity was evaluated using an Agilent Bioanalyzer 2100. Samples with an RNA integrity number (RIN) > 7.0 were used for library preparation. Poly(A)+ RNA was enriched, fragmented, and reverse-transcribed to generate strand-specific cDNA libraries. The final libraries were sequenced using paired-end 150 bp sequencing (PE150) on an Illumina NovaSeq 6000 platform (LC-Biotechnology Co., Ltd., Hangzhou, China). RNA-seq was performed using three independent biological replicates per group.

For bioinformatics analysis, raw reads were filtered using Cutadapt (version cutadapt-1.9) to remove adaptor-containing reads, polyA/polyG reads, reads containing more than 5% unknown nucleotides, and low-quality reads. Sequence quality was assessed using FastQC (version 0.11.9). Clean reads were aligned to the *Homo sapiens* reference genome (GRCh38) using HISAT2 (version 2.2.1). Differential expression analysis between groups was performed using DESeq2 (version 1.22.2), and genes with a false discovery rate (FDR) < 0.05 and absolute fold change ≥ 2 were considered differentially expressed genes. Differentially expressed genes were subsequently subjected to Gene Ontology (GO) and Kyoto Encyclopedia of Genes and Genomes (KEGG) enrichment analyses.

### 2.12. qPCR Analysis of DEGs

Total RNA was extracted using TRIzol reagent (Invitrogen, USA) according to the manufacturer’s instructions. cDNA was synthesized from total RNA using the PrimeScript™ RT reagent Kit with gDNA Eraser (RR047A; Takara Bio Inc., Kusatsu, Shiga, Japan). Quantitative real-time PCR (qPCR) was performed to validate the expression of selected differentially expressed genes (DEGs) and verify the consistency of the two comparisons, using TransStart^®^ Tip Green qPCR SuperMix (TransGen Biotech, Beijng, China) on a CFX Opus 96 Real-Time PCR System (Bio-Rad, Hercules, CA, USA) according to the manufacturer’s instructions. The amplification conditions were as follows: initial denaturation at 94 °C for 30 s, followed by 45 cycles of 94 °C for 5 s and 60 °C for 30 s. The primer sequences were as follows: β-actin, forward 5′-TATGGAATCCTGTGGCATC-3′ and reverse 5′-GTGTTGGCATAGAGGTCTT-3′; FST, forward 5′-ACGTGTGAGAACGTGGACTG-3′ and reverse 5′-CACATTCATTGCGGTAGGTTTTC-3′; AKR1C2, forward 5′-TTCCAGTGTCTGTAAAGGAGGA-3′ and reverse 5′-CTTGTAGACATGCAATCACGGA-3′; JDP2, forward 5′-CTCTCAGTCTTGGGGCCTTC-3′ and reverse 5′-CCAGGCATCATAGCAGGAGG-3′; and KLF9, forward 5′-GCCGCCTACATGGACTTCG-3′ and reverse 5′-GCCGTTCACCTGTATGCAC-3′. Relative gene expression was normalized to β-actin and calculated using the 2^−ΔΔCt^ method.

### 2.13. Statistical Analysis

Quantitative experiments were performed using three independent biological replicates. Data are presented as the mean ± standard deviation (SD). Statistical analyses were performed using GraphPad Prism (version 10.3.0). Data distribution was assessed for normality using the Shapiro–Wilk test. For normally distributed data with sufficient sample size, comparisons between two groups were performed using an unpaired Student’s *t*-test, while for small samples or non-normally distributed data (e.g., Figures 3C and 4A), the Mann–Whitney U test was applied. Multiple-group comparisons were analyzed using two-way ANOVA followed by appropriate post hoc tests. Flow cytometry data were analyzed with ModFit LT (version 5.0). For RNA-seq, differentially expressed genes were identified using DESeq2, and genes with an FDR < 0.05 and absolute fold change ≥ 2 were considered differentially expressed. All tests were two-tailed, and a *p* value < 0.05 was considered statistically significant. The levels of statistical significance were denoted as follows: * *p*  <  0.05, ** *p*  <  0.01, *** *p* < 0.001, **** *p* < 0.0001 and ns (not significant).

## 3. Results

### 3.1. Overexpression of MEOX1 Reduces Cell Proliferation and Increases the G2/M Phase Fraction in SACC Cells

**Figure 1:**
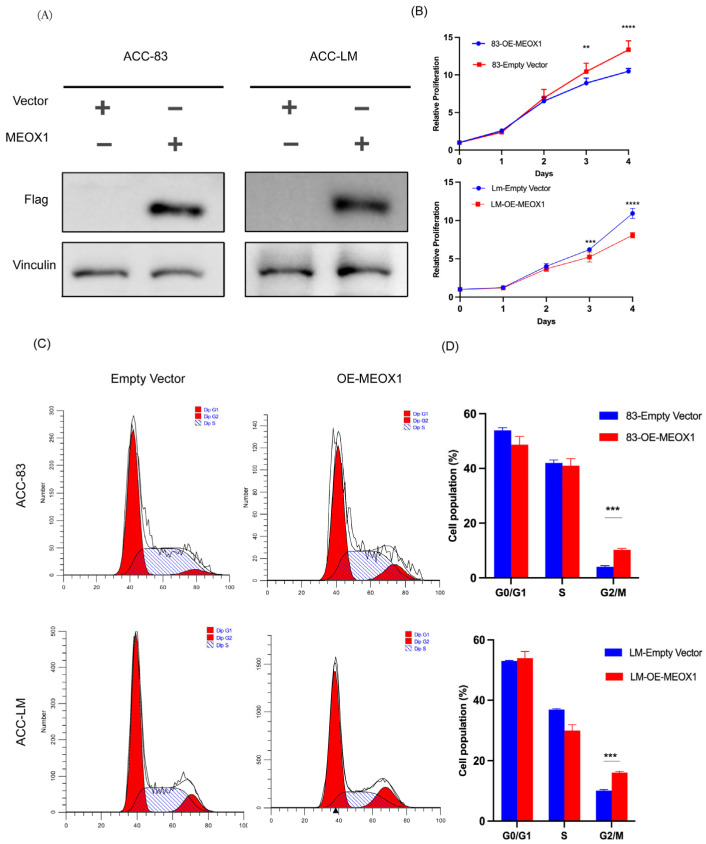
Overexpression of MEOX1 reduces cell proliferation and increases the G2/M phase fraction in SACC cells. (**A**) Western blot analysis confirming MEOX1 overexpression in SACC-83 and SACC-LM cells. (**B**) The CCK-8 assay showing cell proliferation at the indicated time points. Data are presented as the mean ± SD from three independent biological replicates, each with five technical replicates per condition. Statistical analysis was performed using two-way ANOVA. (**C**) Flow cytometric analysis of cell cycle distribution in control and MEOX1-overexpressing SACC cells. (**D**) Quantitative analysis of the cell cycle distribution shown in (C), including the percentages of cells in the G0/G1, S, and G2/M phases. Three independent biological replicates were performed. Data are presented as the mean ± SD. * *p* < 0.05, ** *p* < 0.01, *** *p* < 0.001, **** *p* < 0.0001 and ns (not significant).

Given the context-dependent roles of MEOX1 reported across different tumor types, we first examined its effect in SACC. To investigate this, we generated stable MEOX1-overexpressing SACC-83 and SACC-LM cell lines via lentiviral transduction, and successful overexpression was confirmed by Western blot analysis (Figure 1A). We then assessed the effect of MEOX1 overexpression on cell proliferation using the CCK-8 assay. Compared with their respective control cells, both SACC-83 and SACC-LM cells overexpressing MEOX1 showed reduced proliferative capacity over time (Figure 1B). We next evaluated whether this growth phenotype was accompanied by changes in cell cycle distribution. Flow cytometric analysis showed that MEOX1 overexpression increased the proportion of cells in the G2/M phase in both SACC-83 and SACC-LM cells relative to the control group (Figure 1C,D). This shift was accompanied by a relative decrease in the proportion of cells in the remaining phases. Together, these findings indicate that MEOX1 overexpression is associated with reduced proliferation and an increased G2/M phase fraction in SACC cells.

### 3.2. Overexpression of MEOX1 Impairs Invasion and Migration of SACC Cells In Vitro

**Figure 2:**
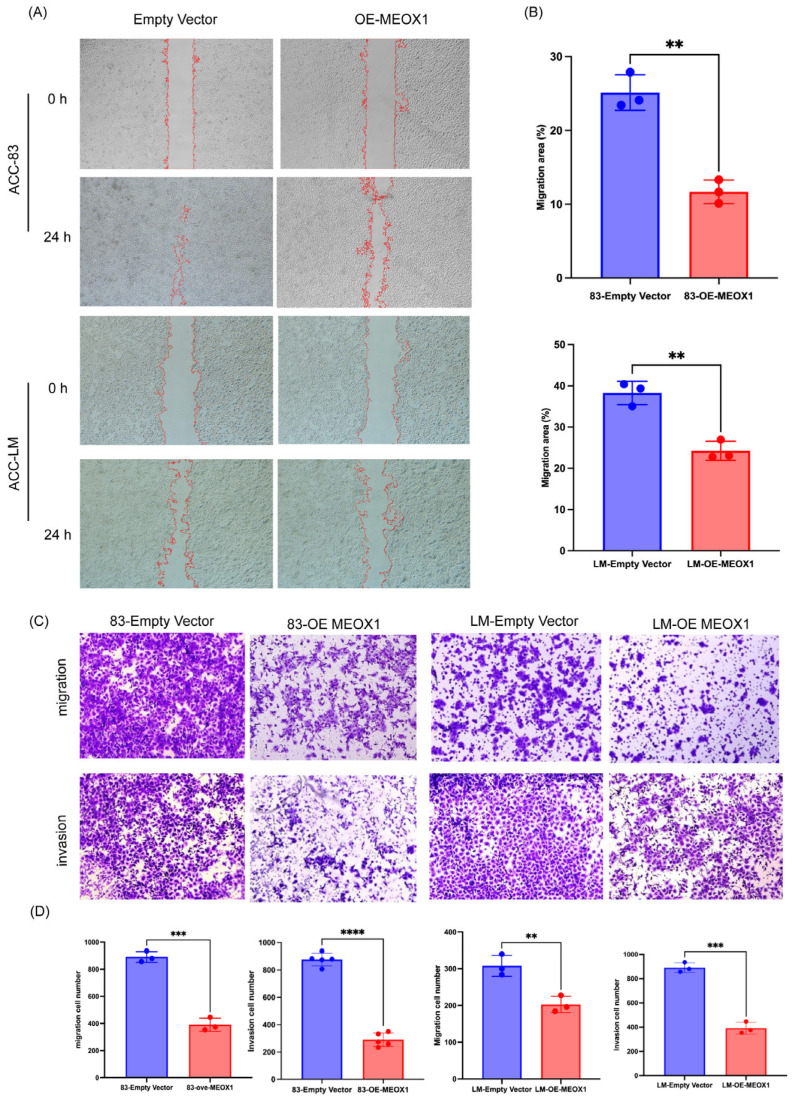
MEOX1 overexpression impairs migration and invasion of SACC cells in vitro. (**A**) Representative images from wound healing assays of control and MEOX1-overexpressing SACC cells at 0 and 24 h. (**B**) Quantification of wound closure rates. Data are presented as the mean ± SD from three independent biological replicates. Statistical analysis was performed using an unpaired Student’s *t*-test. (**C**) Representative images from Transwell migration and invasion assays in control and MEOX1-overexpressing SACC cells. (**D**) Quantification of migrated and invaded cells. Data are presented as the mean ± SD from three independent biological replicates. Statistical analysis was performed using an unpaired Student’s *t*-test. ** *p* < 0.01, *** *p* < 0.001, **** *p* < 0.0001.

We then asked whether MEOX1 overexpression influenced the motile and invasive behavior of SACC cells. In wound healing assays, MEOX1-overexpressing SACC-83 and SACC-LM cells exhibited delayed wound closure at 24 h relative to their respective control cells, consistent with reduced migratory capacity (Figure 2A,B). This pattern was observed in both cell lines.

The effect of MEOX1 overexpression on cell motility was further assessed using Transwell assays. In both migration and invasion settings, fewer MEOX1-overexpressing cells traversed the membrane than control cells (Figure 2C,D). Similar reductions were detected in both SACC-83 and SACC-LM cells. Collectively, these data indicate that MEOX1 overexpression is associated with impaired migration and invasion of SACC cells in vitro.

### 3.3. MEOX1 Overexpression Suppresses Tumor Growth and Pulmonary Metastatic Colonization In Vivo

**Figure 3:**
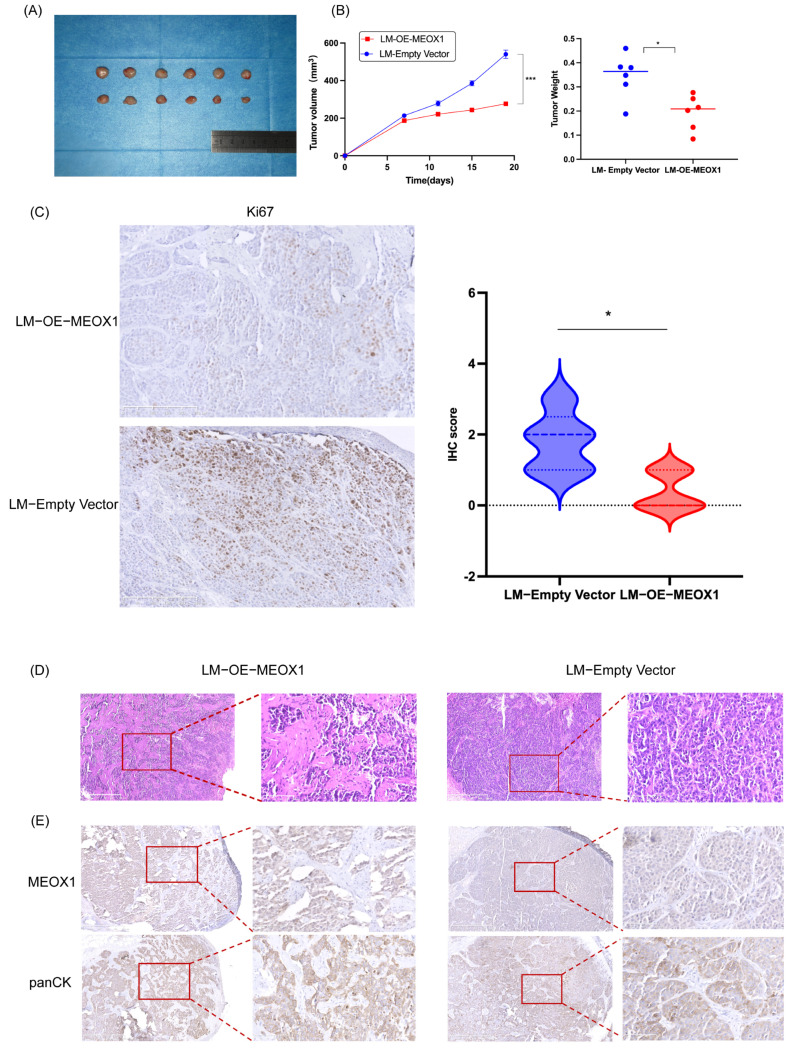
MEOX1 reduces tumor growth and alters histological features in xenograft models. (**A**) Representative images of xenograft tumors from the empty vector and OE-MEOX1 groups. (**B**) Tumor growth curves and endpoint tumor weights of xenografts derived from control or MEOX1-overexpressing SACC-LM cells. Mice were subcutaneously injected with 5 × 10^6^ cells, and each group included six mice *(n* = 6). Data are presented as the mean ± SD. Statistical analysis was performed using two-way ANOVA for tumor growth curves and an unpaired Student’s *t*-test for endpoint tumor weight. (**C**) Representative immunohistochemical staining for Ki67 in formalin-fixed xenograft tumor sections, with violin plots showing semi-quantitative IHC scores based on the percentage of positively stained tumor cells. Only tumor cells were scored. Group comparison was performed using the Mann–Whitney U test. Scale bar = 50 μm. (**D**) Representative hematoxylin and eosin (H&E) staining of xenograft tumor sections showing the overall histological architecture. Scale bar = 50 μm. (**E**) Representative immunohistochemical staining for MEOX1 and pan-cytokeratin (pan-CK) in xenograft tumor tissues. Scale bar = 50 μm. * *p* < 0.05, *** *p* < 0.001.

To determine whether the inhibitory effects observed in vitro could also be detected in vivo, we first established a subcutaneous xenograft model using SACC-LM cells stably overexpressing MEOX1 or the empty vector. Tumors derived from MEOX1-overexpressing cells grew more slowly than those in the control group over the course of observation (Figure 3A,B). At the endpoint, tumor weight was lower in the MEOX1-overexpressing group than in the control group (*n* = 6; Figure 3B).

Ki67 immunohistochemical staining was performed to further assess proliferative activity in xenograft tissues. Tumors from the MEOX1-overexpressing group showed lower Ki67 staining scores than tumors from the control group (Figure 3C). Histological examination by H&E staining further showed morphological differences between the two groups. Compared with the control group, tumors derived from MEOX1-overexpressing cells displayed reduced solid epithelial tumor areas and increased stromal regions (Figure 3D). In addition, immunohistochemical staining for MEOX1 and pan-CK showed that MEOX1 expression was localized to epithelial tumor cells and was not detected in stromal regions (Figure 3E).

**Figure 4:**
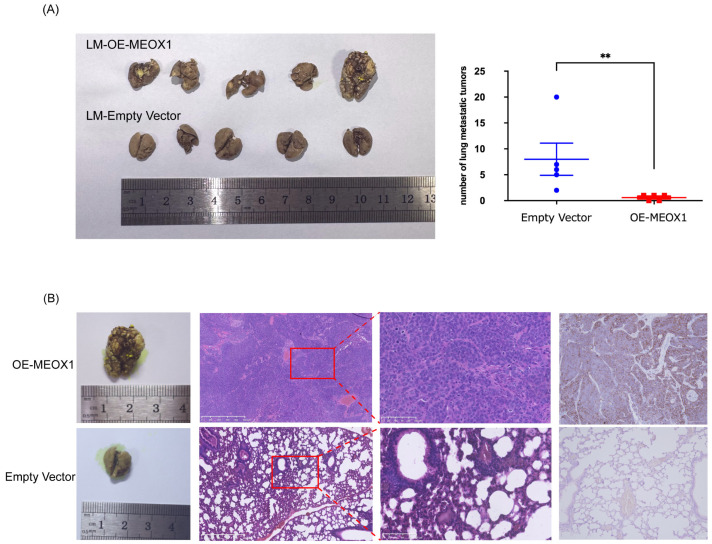
MEOX1 overexpression reduces pulmonary metastatic colonization in an experimental lung metastasis model. (**A**) Quantification of pulmonary metastatic nodules in mice injected intravenously with OE-MEOX1- or empty vector-transduced SACC-LM cells. Five mice per group were included in the final analysis. Data are presented as the mean ± SD. Group comparison was performed using the Mann–Whitney U test. (**B**) Representative hematoxylin and eosin (H&E) staining of lung sections showing metastatic tumor nodules. Immunohistochemical staining for pan-cytokeratin (pan-CK) was performed to identify epithelial tumor cells. Scale bar = 50 μm. ** *p* < 0.01.

We next used a tail vein injection model to assess pulmonary metastatic colonization. Mice injected with OE-MEOX1 SACC-LM cells developed fewer metastatic pulmonary nodules than mice injected with control cells (Figure 4A). Histological analysis of lung tissues by H&E staining showed extensive tumor infiltration in the control group, accompanied by necrosis and disruption of alveolar structures (Figure 4B). In contrast, lung sections from the OE-MEOX1 group largely retained their alveolar architecture, with only limited tumor involvement observed in the examined sections (Figure 4B). Together, these findings show that MEOX1 overexpression is associated with reduced tumor growth and decreased pulmonary metastatic burden in vivo.

### 3.4. RNA-Seq Reveals MEOX1-Associated Gene Expression Changes

**Figure 5:**
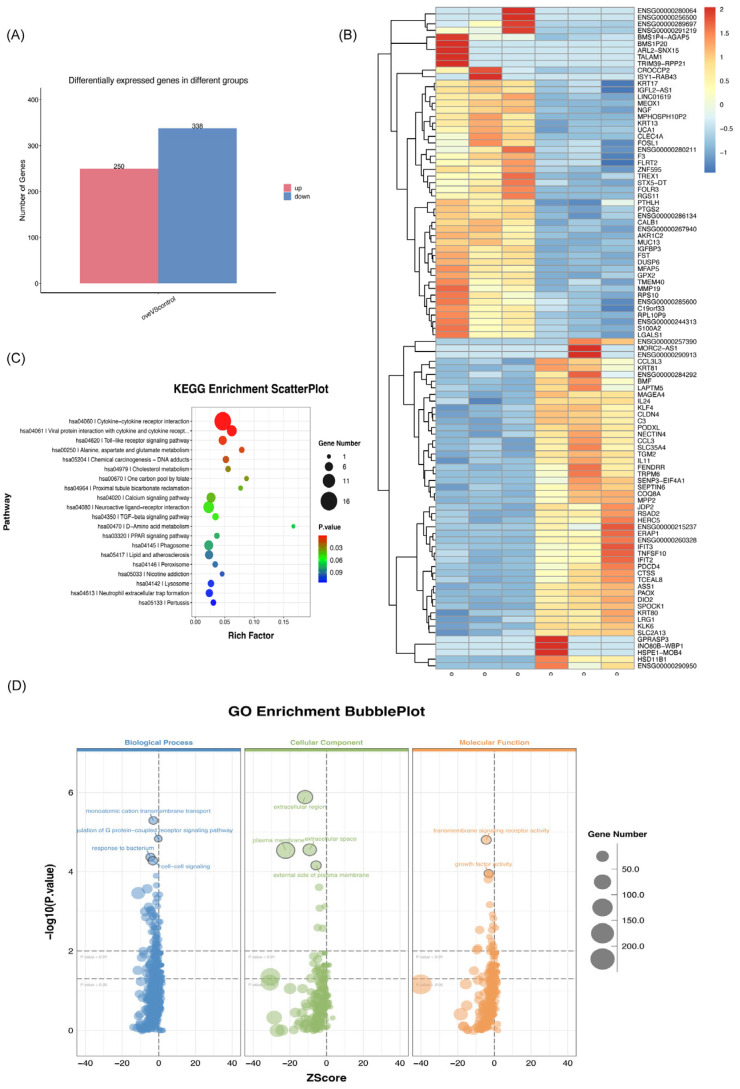
Transcriptomic analysis of MEOX1-overexpressing SACC-83 cells by RNA sequencing. (**A**) Numbers of upregulated and downregulated genes identified by RNA-seq in MEOX1-overexpressing versus control SACC-83 cells (FDR < 0.05, absolute fold change ≥ 2). (**B**) Heatmap of representative differentially expressed genes in control and MEOX1-overexpressing SACC-83 cells. (**C**) KEGG pathway enrichment analysis of differentially expressed genes identified by RNA-seq. (**D**) GO enrichment analysis of upregulated genes in MEOX1-overexpressing SACC-83 cells.

To characterize the transcriptional changes associated with MEOX1 overexpression, we performed RNA-seq analysis in SACC-83 cells with or without MEOX1 overexpression. Differential expression analysis identified a total of 588 differentially expressed genes (DEGs), including 250 upregulated genes and 338 downregulated genes in the MEOX1-overexpressing group relative to the control group (Figure 5A,B).

Functional enrichment analysis was then carried out using the identified DEGs, including KEGG pathway enrichment analysis. The enriched pathways included cytokine–cytokine receptor interaction, Toll-like receptor signaling, chemical carcinogenesis, glutamate metabolism, and alanine, aspartate, and glutamate metabolism (Figure 5C). In parallel, GO enrichment analysis of the upregulated genes showed enrichment in terms related to extracellular signaling and receptor-associated activity, including positive regulation of G protein-coupled receptor signaling and transmembrane receptor activity (Figure 5D). qPCR validation was then performed using two upregulated genes and two downregulated genes, and the results were consistent with the expression trends observed in the RNA-seq data (Supplementary File S1). RNA-seq therefore identified a broad set of transcriptional alterations accompanying MEOX1 overexpression in SACC-83 cells.

## 4. Discussion

SACC is characterized by relatively slow primary tumor growth but a persistent risk of late distant metastasis, a clinical pattern that substantially shapes patient outcomes [2,15]. Defining the biological basis of this apparent dissociation between limited proliferative kinetics and marked metastatic propensity is therefore of considerable clinical relevance. In the present study, gain-of-function models consistently showed that enforced MEOX1 overexpression was associated with a less aggressive phenotype in SACC. In both SACC-83 and SACC-LM cells, MEOX1 overexpression reduced proliferative capacity, increased the G2/M phase fraction, and impaired migration and invasion in vitro. These observations were further supported in vivo, where MEOX1-overexpressing xenografts displayed reduced tumor growth, lower Ki67 staining, and altered histological architecture characterized by decreased epithelial tumor areas and more prominent stromal regions. Notably, MEOX1 staining was confined to epithelial tumor cells and was not detected in the stromal compartment, suggesting that the stromal changes observed in xenografts were more likely secondary to tumor cell-associated effects than to direct stromal expression of MEOX1.

This histological shift is of particular interest in light of prior studies implicating MEOX1 in fibrotic tissue remodeling in the heart and lungs, where it has been linked to fibroblast activation and extracellular matrix deposition in association with TGF-β1 signaling [10,11,16,17]. Although these observations derive primarily from non-neoplastic settings, fibrotic and matrix remodeling programs are increasingly recognized as integral components of the tumor microenvironment. In this context, our findings raise the possibility that MEOX1 overexpression in SACC cells may be associated not only with altered tumor cell behavior but also with secondary changes in stromal organization.

Transcriptomic profiling provided additional clues to pathways associated with MEOX1 overexpression. Differentially expressed genes were enriched in pathways related to cytokine–cytokine receptor interaction, Toll-like receptor signaling, G protein-coupled receptor signaling, and other receptor- or extracellular signaling-associated processes. Although enrichment analyses do not establish mechanism, these patterns suggest that MEOX1 overexpression may be accompanied by broader changes in environmental sensing, intercellular communication, adhesion, and motility. In line with reports from lung cancer that link MEOX1 to G2 arrest via repression of CCNB1 [18], it will be important in future studies to determine whether MEOX1 directly influences canonical cell cycle regulators in SACC, including molecules governing the G2/M transition. Likewise, the enrichment of cytokine- and GPCR-related pathways nominates candidate signaling modules for follow-up in co-culture, conditioned medium, and perturbation-based experiments.

Available evidence indicates that the function of MEOX1 is strongly context-dependent. In several malignancies, including HER2-positive and triple-negative breast cancer, glioblastoma, and ovarian cancer, MEOX1 has been associated with aggressive phenotypes and adverse clinicopathological features, in some settings acting downstream of PBX family regulators [12,19,20,21,22,23,24]. In contrast, studies on subsets of non-small-cell lung cancer have suggested growth-restrictive effects and associations with more favorable outcomes [5,18,25,26]. Such bidirectional behavior may reflect lineage-specific transcriptional networks, distinct chromatin states, co-occurring genomic alterations, and differences in transcription factor partners. Our findings extend this context-dependent model to SACC and suggest that, in gain-of-function settings, MEOX1 overexpression is associated with attenuation of malignant phenotypes in this disease.

This tumor-restraining pattern is also consistent with a broader, although still incomplete, body of evidence suggesting that a loss of suppressive regulators is an important component of SACC progression. At the genomic level, recurrent loss of chromosome 6 has long implied the presence of tumor-suppressive loci in ACC. However, although PLAGL1 and LATS1 were initially prioritized as candidate genes within the commonly deleted region, subsequent mutation and expression analyses failed to identify either as the definitive target, highlighting the genetic complexity of tumor suppressor inactivation in this disease [27]. More recent studies have instead drawn attention to post-transcriptional and signaling-mediated suppressive mechanisms. Several microRNAs have been shown to exert clear tumor-suppressive effects in SACC. miR-98 inhibits proliferation, migration, and invasion by targeting N-RAS and attenuating PI3K/AKT and MAPK/ERK signaling [28]. miR-144-3p suppresses proliferation and promotes apoptosis through direct repression of mTOR signaling [29], miR-5191 restrains proliferation, invasion, and pulmonary metastasis by targeting Notch-2 and downregulating c-Myc, Bcl-2, Hes-1, Hey-1, and Cyclin D1 [30]. More recently, miR-429 has been reported to inhibit proliferation, invasion, metastasis, and perineural invasion through ZEB1-dependent suppression of EMT-related programs [31]. In parallel, findings from salivary gland malignancy models indicate that disruption of the tumor-suppressive PP2A axis, as reflected by decreased Ppp2r1b expression and upregulation of endogenous inhibitors such as CIP2A and SET, may promote malignant transformation in adenoid cystic carcinoma, possibly through mTOR pathway activation [32]. Collectively, these findings indicate that SACC progression may result not only from activation of oncogenic drivers, but also from convergent disruption of multiple tumor-suppressive layers, spanning chromosomal integrity, phosphatase signaling, and microRNA-mediated post-transcriptional control. Within this framework, the growth-inhibitory and anti-invasive effects observed here support MEOX1 as a plausible addition to the emerging network of suppressive regulators in SACC.

Beyond tumor cell intrinsic programs, MEOX1 has been linked to broader microenvironmental and immunologic features in other tumor types. For example, MEOX1 expression has been associated with regulatory T-cell enrichment in intrahepatic cholangiocarcinoma and with stromal proliferation and cancer stem cell-related traits in breast cancer [25]. In our study, the altered stromal architecture observed in xenografts raises the possibility that the consequences of MEOX1 overexpression in SACC may extend beyond cell cycle and motility phenotypes. Nonetheless, these observations remain preliminary, and more detailed analysis of stromal composition and signaling will be required before any definitive conclusions can be drawn.

The translational significance of MEOX1 in SACC remains to be determined. Given the limited efficacy of systemic therapies for recurrent or metastatic SACC, the phenotypic and transcriptomic changes observed in our overexpression models suggest that MEOX1-associated pathways may merit further investigation as candidate biological vulnerabilities.

Several limitations of this study should be acknowledged. First, our conclusions are based primarily on gain-of-function models and therefore do not establish whether endogenous MEOX1 is required for the observed phenotypes in SACC. Loss-of-function and rescue experiments will be necessary to define the requirement and specificity of MEOX1 in this context. Second, the direct downstream targets and chromatin occupancy landscape of MEOX1 in SACC remain undefined. Integrative approaches such as ChIP-seq, ATAC-seq, and perturbation-based transcriptomic profiling may help clarify its regulatory circuitry. Third, clinical validation is currently lacking. Analyses of primary SACC specimens will be needed to determine whether endogenous MEOX1 expression varies across histologic or molecular subsets and whether such variation is associated with clinicopathological features or patient outcomes. Of note, EN1 has recently been implicated as a transcriptional regulator and potential therapeutic node in SACC [24]. Motif analysis using the JASPAR database suggests a possible interaction between MEOX1 and EN1, raising the possibility of a cooperative or hierarchical regulatory relationship that warrants further investigation.

## 5. Conclusions

In summary, our data show that enforced MEOX1 overexpression is associated with less aggressive phenotypes in SACC models, together with G2/M cell cycle accumulation and transcriptomic alterations in multiple cancer-related pathways. These observations expand the current understanding of MEOX1-associated changes in SACC and provide a basis for future mechanistic studies.

## Data Availability

The raw data supporting the conclusions of this article will be made available by the authors on request.

## Supplementary Materials

The following supporting information can be downloaded at: https://www.mdpi.com/article/10.3390/cimb48050485/s1.

## Abbreviations

The following abbreviations are used in this manuscript:

SACC	Salivary adenoid cystic carcinoma
MEOX1	Mesenchyme homeobox 1
GPCR	G protein-coupled receptor
FBS	Fetal bovine serum
PBS	Phosphate-buffered saline
IHC	Immunohistochemistry
H&E	Hematoxylin and eosin
CCK-8	Cell Counting Kit-8
RNA-seq	RNA sequencing
DEG	Differentially expressed gene
GO	Gene Ontology
KEGG	Kyoto Encyclopedia of Genes and Genomes
OE	Overexpression
SD	Standard deviation
TME	Tumor microenvironment
ANOVA	Analysis of variance
NIH	National Institutes of Health
