# Supplementary material for: MEOX1 Inhibits Growth and Metastasis of Salivary Adenoid Cystic Carcinoma
Source: Curr Issues Mol Biol. 2026 May 6;48(5):485. doi: 10.3390/cimb48050485 (PMC13204662; doi:10.3390/cimb48050485)
Supplement: Supplementary file 1 [file cimb-48-00485-s001.zip › Supplementary File S1.pdf]

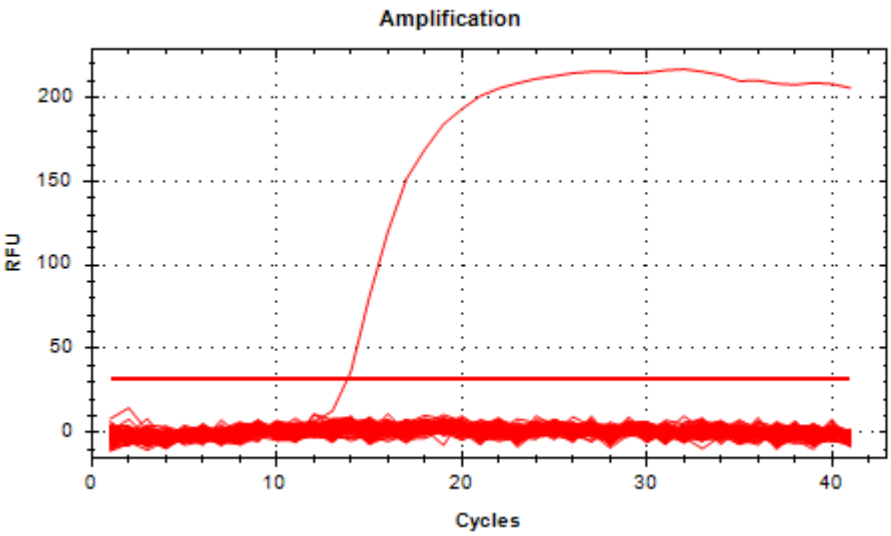

Amplification curves of the mycoplasma qPCR assay.

The positive control exhibited a typical sigmoidal amplification curve, whereas no amplification signal was detected in the tested sample(s), supporting the absence of detectable mycoplasma contamination.
